# E-Bikes (Electrical Bicycles and Scooters) Related Neurosurgical Injuries in the Adult Population: A Single-Center Experience

**DOI:** 10.1089/neur.2023.0071

**Published:** 2023-11-16

**Authors:** Carla Richetta, Yevgeny Karepov

**Affiliations:** Department of Neurosurgery, Tel-Aviv Sourasky Medical Center, Tel-Aviv, Israel.

**Keywords:** adults, e-bikes, helmet, neurosurgery, traumatic brain injury

## Abstract

E-bikes (electrical bicycles and scooters) have been increasingly used as a means of transportation, especially among young adults. E-bikers have more accidents, at higher velocities and more severe kinematics, increasing the rate of neurosurgical injuries. Severe neurosurgical injury patterns result in significant morbidity and mortality. We collected data regarding adult patients (>18 years old), who suffered e-bike-related neurosurgical injuries, in a single tertiary medical center in Israel, between July 2019 and June 2020. Fifty-eight consecutive patients were included in this study. The average age was 34.9, and the average Glasgow Coma Scale (GCS) score upon admission was 13.2 and was significantly lower in operated patients (10.75). Fifty-four patients were riders; 51 (94.5%!) were not wearing a helmet. Fifty percent of patients had multiple types of trauma. Six patients suffered a spinal injury. Sixteen patients required either cranial or spinal surgery. Three patients died, and 1 remained in a vegetative state. Median Glasgow Outcome Scale-Extended (GOS-E) score at follow-up was 7.1. Operated patients stayed significantly longer in the intensive care unit (ICU) and in the hospital, and their GOS-E scores at discharge and follow-up were significantly lower. Most spinal injuries underwent surgery. Patients who wore helmets had significantly higher GCS scores and a shorter stay in the ICU and hospital. The unacceptable reality of the careless use of this transportation and the unique kinematics lead to severe neurosurgical injuries, comorbidities, and even mortality. Our results reflect the risks of e-bikes in the adult population. Most of our patients were in the mid-age group, and almost none had used a helmet. The results of this study highlight the potential need for neurosurgical treatment, and the need for long-term rehabilitation and follow-up, reflecting the emotional and financial toll of these injuries. Once again, this study showed that helmets save lives and emphasized the importance of protecting our heads.

## Introduction

Electrical bicycles and scooters (e-bikes) are a new rising star in the field of modern urban transportation. E-bikes are light electrical vehicles, highly available for rent and easy to operate, making it an attractive type of transportation, especially for the younger population.^1–6^

E-bikes provide an attractive type of transportation.^2–4^ Currently, e-bikes are the most widely used electrical transportation in the world. There is no world-wide cohesive legislation regarding e-bikes, nothing unified defining the minimal user's age, nor any type of e-bike power and speed limitations. Every modern country legislates its own special laws regarding the use of e-bikes.^1,2,6–8^

Even though e-bikers should ride only on the road, they can be found riding wildly on the sidewalks, jeopardizing both pedestrians and themselves.^2,4,6,8^

The same situation can be found on the roads, while e-bike users are executing dangerous maneuvers (i.e., crossing roads diagonally), often on red lights, and ignoring most of the traffic rules.^1,3,4,7,9^

E-bike-related injuries were described recently in adults and children,^2,3,5,6,8,10^ whereas the adult population constitutes two thirds of overall e-bike trauma (65%),^1,2,6,8^ leading mostly to orthopedic trauma, traumatic brain injury (TBI), and neck trauma.^5–8,10^

When we compare e-bikers to regular bicyclists, the first group has been found to have more accidents (falls; collisions with static objects, pedestrians, or other vehicles), at higher velocities and more sever collision kinematics.^2,5–7,10^

Thus, e-bike-related injuries result in more severe morbidity and mortality, both for direct users and pedestrians caught up in a horrifying situation.^1,5,9,10^ These injuries have patterns that resemble motorcycle-related injuries, compared to the injuries of regular bicyclists.^2,4–7,9,10^

The use of e-bikes in Israel has risen dramatically in recent years, often by teenagers and young adults, without sufficient education regarding traffic rules. These users often neither respect traffic rules, nor use personal protective gear, especially helmets.^2,6–8^

Recent studies showed that wearing a helmet reduces TBI by 50%.^11,12^

The combination of easily accessible and convenient transportation with increased risk for more severe injury patterns, and the fact that most of the users do not wear helmets, make e-bikers much more susceptible to neurosurgical injuries.^1,3–6,8,9^

The aim of this study was to evaluate e-bike-related cranial and spine injuries in the adult population and describe our single-center experience treating e-bike-related neurosurgical injuries.

## Methods

Subsequent to an institutional review board (IRB) approval, data were retrospectively collected, on all e-bike-related neurosurgical injuries admitted to the Emergency Department at Tel-Aviv Sourasky Medical Center (Tel-Aviv, Israel) between July 2019 and June 2020.

All patients included in the study were >18 years old at the time of admission. The collected data consisted of demographics, mechanism of trauma (i.e., fall from e-bike, collide with objects, or collide with other vehicle), type of e-bike user (i.e., whether the patient rode on the e-bike, was mounted on, or was injured as a pedestrian), other accompanying body injuries, use of a helmet, Glasgow Coma Scale (GCS) at admission, underlying neurosurgical injury, cranial and cervical imaging findings, days in the intensive care unit (ICU; neurosurgical ICU [NICU] or general ICU), neurosurgical interventions, duration of hospital stay (DOS), and neurological outcome (Glasgow Outcome Scale-Extended; GOS-E) at discharge and follow-up. Motorcycle and non-electrical bicycle or scooter injuries were excluded from the study.

TBI or spinal trauma without radiological findings were excluded, as well as patients with missing radiological findings or clinical data regarding the mechanism of accident or clinical course.

Data were extracted from personal folders and follow-up notes, a local server (NeuroSurgery), and hospital database software (Chameleon, NAMER, and ADA). Imaging data were retrieved from an imaging server (PACS).

Patient and family consents were waived by the IRB.

### Statistical analysis

Most of the data are presented in a descriptive manner, and on some data, basic statistics were performed (mean ± standard deviation).

To determine the significance in difference of means involving independent samples, we used an unpaired two-tailed Student *t*-test; *p* < 0.05 was considered significant.

To identify the significance of risk factors for independent populations without a normal distribution population, we used a two-sample rank test (the Mann-Whitney U test); *p* < 0.05 (two-tailed) was considered significant.

Statistical analysis was performed with IBM SPSS Statistics software (version 28; IBM Corp., Armonk, NY).

## Results

### General data

Fifty-eight e-bike-related neurosurgical trauma patients were included in this study. Forty-five (77.6%) were male; average age was 34.9 ± 14.6 years (range, 18–73).

Fifty-four patients were riders, and 51 (94.5%) were not wearing a helmet!

Four patients were hit as pedestrians; all 4 were hit by scooters. Among riders, 21 were e-bicycle riders and 33 used electrical scooters.

Average GCS upon admission to the emergency room (ER) was 13.2 ± 3.41. Three patients among riders (*n* = 3 of 54; 5.5%) wore a helmet; all had GCS 15 at admission.

Patients were hospitalized in the ICU for 2.34 days on average, and 8.5 days was the mean DOS in the hospital.

### Mechanism and types of injury

The main mechanisms of injury were fall off an e-bike (40 patients; 68%) and collision with another vehicle (14 patients; 24%).

The most common injuries were skull fractures (*n* = 32; 55%), whereas 20 of skull fractures (20 of 32; 62.5%) involved frontal bone.

Most patients suffered from intracranial bleeding, presented accordingly: acute subarachnoid hemorrhage (SAH; *n* = 20; 34%); intraparenchymal contusions (intracerebral hemorrhage [ICH]; *n* = 17; 29%); acute subdural hematoma (ASDH; *n* = 15; 26%); and acute epidural hematoma (EDH; *n* = 10; 17%).

In half of the cases, multiple types of traumas were combined (*n* = 29; 50%). Six patients (10%) suffered a spinal fracture; One of these patients had a cord contusion.

### Surgeries

Sixteen of 58 patients (27.6%) required either cranial or spinal surgery because of their injuries: Six underwent a decompressive hemicraniectomy; Four underwent spinal surgery, and two underwent insertion of an intracranial pressure (ICP) monitor. Three patients underwent maxillofacial surgery, and 1 patient underwent plastic surgery for laceration debridement and closure.

Subsequently, 5 patients underwent a cranioplasty (4 autologous bone and 1 Matrix Mesh), and 1 patient underwent the insertion of a V-P shunt as developments of post-traumatic hydrocephalus.

### Patients' outcome at discharge and long-term follow-up

Three patients died (5%), 1 patient (1.7%) remained in a vegetative state, 3 patients (5%) were severely disabled, and 3 patients (5%) were moderately disabled. Two patients (3.4%) recovered well, and 46 patients (79%) were discharged intact.

GOS-E at discharge and follow-up was 7.12 ± 2.00 and 7.42 ± 1.10, respectively. Patients were followed for a median of 11.5 ± 12.4 months. Demographics and general data are displayed in Table 1.

**Table 1.** General Demographics

	**58 patients**
Age, median ± std	34.9 ± 14.6
Range	18–73
Sex (%)	
Male	45 (77.6)
Female	13 (22.4)
GCS at admission, median ± std	13.20 ± 3.41
Use of helmet (%)	3/54 (5.5)
Mechanism of trauma (%)	
Fall from e-bike/object	40 (69)
Collided with another vehicle	14 (24)
Pedestrian	4 (7)
Type of e-bike (%)	
e-bicycles	21 (36)
e-scooter	37 (64)
Type of trauma (%)	
Skull fracture	32 (55)
ASDH	15 (26)
SAH	20 (34)
EDH	10 (17)
ICH	17 (29)
Spinal trauma	6 (10)
Multi-trauma	29 (50)
Days in ICU, median ± std	2.34 ± 5.25
DOS in the hospital, median ± std	8.50 ± 15.34
Follow-up time, median ± std	11.5 ± 12.4
GOS-E at discharge, median ± std	7.12 ± 2.00
GOS-E at follow-up, median ± std	7.42 ± 1.10
Surgery (%)	16 (27.6)
Decompressive craniectomy	6 (10.3)
ICP monitor insertion	2 (3.4)
Spinal surgery	4 (6.8)
Maxillofacial or plastic	4 (6.8)

std, standard deviation; GCS, Glasgow Coma Scale; ASDH, acute subdural hematoma; SAH, subarachnoid hemorrhage; EDH, epidural hematoma; ICH, intracerebral hemorrhage; ICU, intensive care unit; DOS, duration of stay; GOS-E, Glasgow Outcome Scale-Extended; ICP, intracranial pressure.

In the operated patients' group, mean GCS at admission was significantly lower in comparison to non-operated patients (10.75 ± 4.60 vs. 14.1 ± 2.3; *p* = 0.006, accordingly); these patients stayed for significantly more days in the ICU and hospital (5.25 ± 7.10, *p* = 0.046 and 19.4 ± 22.9, *p* = 0.024, accordingly). GOS-E at discharge and follow-up was significantly lower (5.7, *p* = 0.013 and 6.8, *p* = 0.041, respectively) in comparison to non-operated patients. Operated patients were also followed up for much longer (19.5 ± 13.8 vs. 8.4 ± 10.4 months; *p* = 0.0134), and none were lost to follow-up. Five non-operated patients were lost to follow-up after discharge from the hospital.

### Other injury risk factors

Spinal trauma was the only mechanism of injury that was found as a risk factor in the operated patients' group (*p* = 0.023, relative risk [RR] = 5.25). None of the pedestrians were operated (0%; *p* = 0.044, RR = 0.105).

A comparison of operated vs. non-operated patients is shown in Table 2.

**Table 2.** Surgical vs. Non-Surgical

	**Non-surgical**	**Surgical**	*p* **value**
	***42***	***16***	
Age, median ± std	35.20 ± 5.05	34.2 ± 13.8	0.81
Range	18–73	21–70	
Sex (%)			
Male	31 (71.4)	14 (87.5)	0.11
Female	11 (28.6)	2 (12.5)	
GCS at admission, median ± std	14.1 ± 2.3	10.75 ± 4.60	0.006
Use of helmet (%)	2/38 (5)	1/16 (6.2)	0.44
Use of helmet cranial injury (%)	2/36 (5.6)	0/12 (0)	0.08
Mechanism of trauma (%)			
Fall from e-bike/object	27 (64)	13 (81)	0.11
Collided with another vehicle	11 (26)	3 (19)	0.54
Pedestrian	4 (9.5)	0	0.044
Type of e-bike (%)			
e-bicycles	13 (31)	8 (50)	0.09
e-scooter	29 (69)	8 (50)	
Type of trauma (%)			
Skull fracture	25 (59.5)	7 (44)	0.30
ASDH	9 (21)	6 (37.5)	0.22
SAH	15 (28.5)	5 (31)	0.844
EDH	8 (19)	2 (12.5)	0.54
ICH	11 (26)	6 (37.5)	0.41
Spinal trauma	2 (5)	4 (25)	0.023
Multi-trauma	19 (45)	10 (62.5)	0.12
Days in ICU, median ± std	1.24 ± 3.90	5.25 ± 7.10	0.046
DOS in hospital, median ± std	4.36 ± 7.60	19.4 ± 22.9	0.024
Follow-up time, median ± std	8.4 ± 10.4	19.5 ± 13.8	0.0134
GOS-E at discharge median ± std	7.67 ± 1.30	5.70 ± 2.75	0.0129
GOS-E at follow-up median ± std	7.67 ± 0.63	6.78 ± 1.72	0.0409

std, standard deviation; GCS, Glasgow Coma Scale; ASDH, acute subdural hematoma; SAH, subarachnoid hemorrhage; EDH, epidural hematoma; ICH, intracerebral hemorrhage; ICU, intensive care unit; DOS, duration of stay; GOS-E, Glasgow Outcome Scale-Extended; ICP, intracranial pressure.

Patients who wore a helmet had significantly better GCS scores at admission (15.00 vs. 12.94, *p* < 0.001) and a shorter stay in the ICU and hospital (0.33 vs. 2.63 days, *p* = 0.009 and 4.33 vs. 9.11, *p* = 0.0367, accordingly). None of these patients needed cranial surgery (1 patient underwent cervical decompression and fixation; *p* < 0.001). These patients did not present subarachnoid and parenchymatic hemorrhages (0% vs. 39% and 0% vs. 33.3% [*p* < 0.001], accordingly). Helmet-wearing patients' data are presented in Table 3.

**Table 3.** Helmet vs. Non-Helmet

	***Non-helmet***	***Helmet***	p ***value***
	***51***	***3***	
Age, median ± std	32.30 ± 1.35	42.00 ± 21.35	0.58
Range	18–73	24–72	
Sex (%)			
Male	41 (80.4)	2 (66.7)	0.15
Female	10 (19.6)	1 (33.3)	
GCS at admission, median ± std	12.94 ± 3.30	15 ± 0	<0.001
Surgery (%)	15 (29.4)	1 (33.3)	0.44
Surgery cranial (%)	12 (23.5)	0 (0)	<0.001
Mechanism of trauma (%)			
Fall from e-bike/object	38 (64.5)	3 (100)	<0.001
Collided with another vehicle	13 (25.5)	0 (0)	
Type of e-bike (%)			
e-bicycles	18 (35.3)	1 (33.3)	0.45
e-scooter	33 (64.7)	2 (66.7)	
Type of trauma (%)			
Skull fracture	28 (54.9)	1 (33.3)	0.27
ASDH	11 (21.6)	1 (33.3)	0.37
SAH	20 (39)	0 (0)	<0.001
EDH	8 (15.7)	1 (33.3)	0.33
ICH	17 (33.3)	0 (0)	<0.001
Spinal trauma	4 (8)	1 (33.3)	0.27
Multi-trauma	25 (49)	1 (33.3)	0.25
Days in ICU, median ± std	2.63 ± 5.80	0.33 ± 0.47	0.009
DOS in hospital, median ± std	9.11 ± 16.90	4.33 ± 1.70	0.0367
Follow-up time, median ± std	11.07 ± 12.87	19.0 ± 13.1	0.404
GOS-E at discharge, median ± std	7.06 ± 1.75	7.00 ± 1.73	0.956
GOSE at follow-up, median ± std	7.44 ± 1.12	6.67 ± 1.15	0.364

std, standard deviation; GCS, Glasgow Coma Scale; ASDH, acute subdural hematoma; SAH, subarachnoid hemorrhage; EDH, epidural hematoma; ICH, intracerebral hemorrhage; ICU, intensive care unit; DOS, duration of stay; GOS-E, Glasgow Outcome Scale-Extended; ICP, intracranial pressure.

### Glasgow Coma Scale at admission and Glasgow Outcome Scale-Extended

**FIG. 1.**
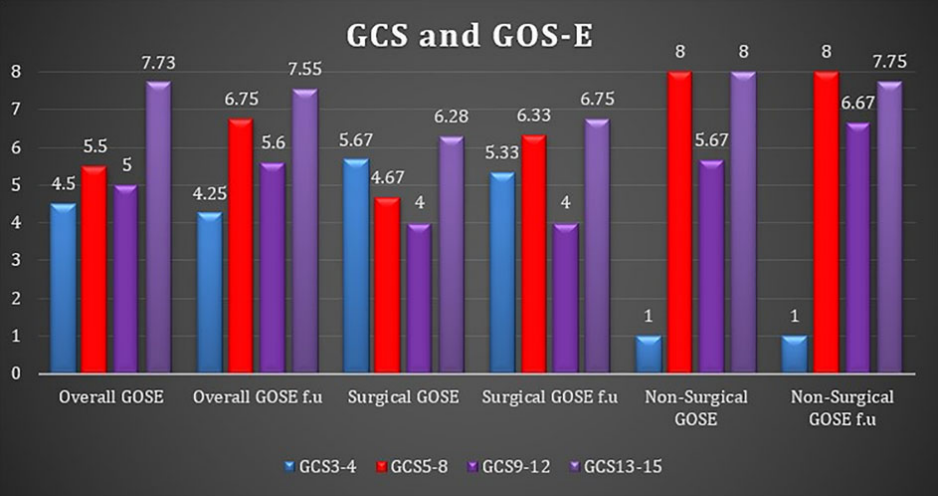
GCS and GOS-E. Bar representation of GOS-E at discharge and follow-up divided to overall, operated patients' group, and non-operated patients. In each group, patients were divided into four subgroups according to severity of injury (GCS). Generally, GOS-E in the operated patients' group was lower than in non-operated patients. The 9–12 GCS group had lower GOS-E than the 5–8 GCS and 13–15 GCS groups. GCS, Glasgow Coma Scale; GOS-E, Glasgow Outcome Scale-Extended.

In the GCS 3–4 group, GOS-E was poor in operated patients, but especially in non-operable patients. In the GCS 13–15 group, outcome was very good, whereas in the GCS 5–8 and 9–12 groups outcome was moderate. This was more prominent in the operated patients. GCS and GOS-E data are presented in Figure 1.

In the long-term follow-up, 5 patients with low GOS-E scores advanced to better clinical performance, whereas proportionally the GOS-E 8 group was smaller at follow-up and the GOS-E 7 group was larger.

Generally, in the long-term follow-up, some patients in the severe and moderate disability groups advanced to the better outcome group. All other groups showed almost no changes in long-term follow-up.

**FIG. 2.**
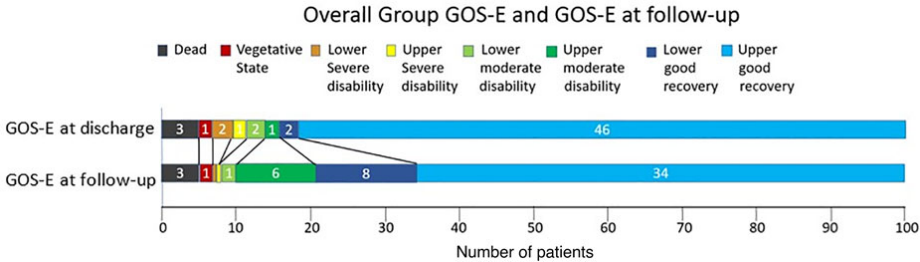
Overall group GOS-E and GOS-E at follow-up. Differentiation and proportion of patients at every GOS-E score. Upper bar indicates GOS-E at discharge, and the lower bar indicates GOS-E at follow-up. We can see that generally more patients have better GOS-E at follow-up, except that already the GOS-E 8 group that had less patients at follow-up. GCS, Glasgow Coma Scale; GOS-E, Glasgow Outcome Scale-Extended.

**FIG. 3.**
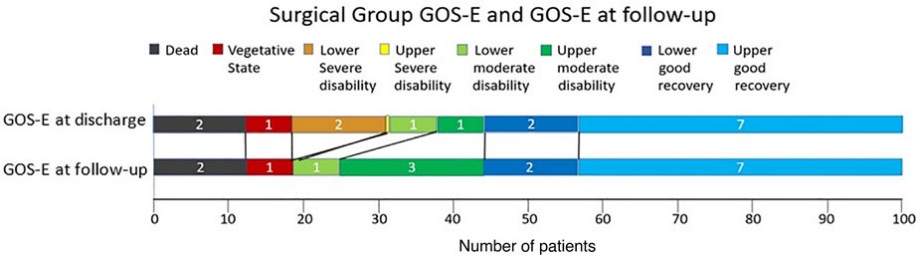
Surgical group GOS-E and GOS-E at follow-up. Proportion of patients at the operated patients' group, at the moment of discharge and at follow-up. Upper bar indicates GOS-E at discharge, and the lower bar indicates GOS-E at follow-up. Generally, there was almost no change in GOS-E, except the GOS-E 5 group, which grew at follow-up. GCS, Glasgow Coma Scale; GOS-E, Glasgow Outcome Scale-Extended.

**FIG. 4.**
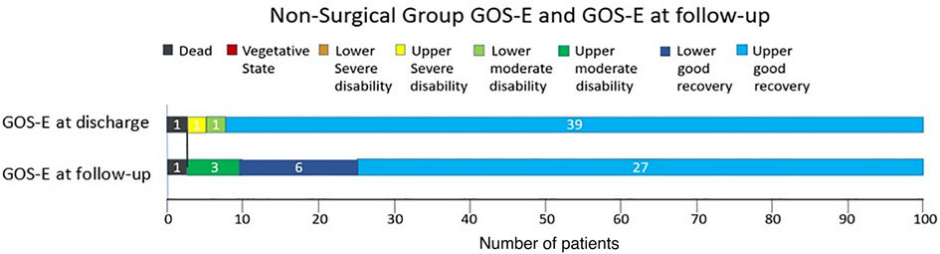
Non-surgical group GOS-E and GOS-E at follow-up. Proportion of patients at the non-operated patients' group, at the moment of discharge and at follow-up. Upper bar indicates GOS-E at discharge, and the lower bar indicates GOS-E at follow-up. The upper good recovery group was smaller at follow-up, but, overall, more patients advanced to a better outcome at follow-up. GCS, Glasgow Coma Scale; GOS-E, Glasgow Outcome Scale-Extended.

Data are shown in Figures 2–4.

## Discussion

E-bikes are increasingly used worldwide.^1–6^ Being relatively cheap, very convenient, and environmentally friendly make this type of transportation very attractive, especially in crowded cities.^1–5^ However, e-bikes carry quite a lot of negative impacts: Secondary to their increased speed, there is an increased potential for injury subsequent to a fall from an e-bike or collision between the e-bike rider and other vehicle or pedestrian.^2,5–7,10^

In recent years, many cities, including Israel, have allocated special areas for bicycle trails. There is >100 km of bike paths just in Tel Aviv. Yet, this solution is not enough, and it proved to be a problematic one as well.

Most e-bikers use regular roads, which leads to e-bike/vehicle conflicts, whereas their use on dedicated regular bicycle paths may lead to e-bike/regular bicycle conflicts.^1–4,8^

E-bikes are vehicles by any means. Despite this fact, to operate an e-bike, one is not obligated to present a driver's license, and there is no world-wide unified legislation regarding e-bikes, nor unified age limits or traffic violations enforcement.^2,6,8,9^

Riding speed can exceed 40 km/h, relying only on battery power. E-bikers perform dangerous maneuvers and traffic violations that lead to potentially severe morbidity and mortality, including to the direct users, other drivers, and pedestrians. There are e-bike users among all age groups.^2,5–9^

Compared to regular bikes, trauma with an e-bike has a unique kinematics, which leads to injury patterns that resemble motorcycle-related injuries.^2,5,7–10^ Many users, especially young ones, underestimate the danger of e-bikes, compared to traditional bicycles.^2,3^

Currently, less than half of e-bikers use helmets, which leads to a significant increase in the risk for moderate-to-severe neurosurgical injuries. The adult population constitutes more than two thirds of e-bikes trauma; thus, it is extremely important to stress the potential risks, especially among this age group.^7–9,11,12^

This study is the first to describe neurosurgical injuries among adults using e-bikes. Almost none of these patients wore a helmet. Most of the patients were young males, while the age range was 18–73 years. Patients who were presented to the ER had mostly mild TBI, with a mean GCS of 13.2. Injury patterns included mostly skull fractures, especially in frontal bone, most probably attributable to an injury mechanism that more resembles motorcycle injuries and not regular bicyclist falls. Approximately half of the injuries were multi-trauma, whereas all types of intra-cranial hemorrhages were represented in our population.

The associated morbidity and mortality are non-negligible, and approximately one quarter of the patients underwent a surgical intervention; 6 patients underwent a decompressive craniectomy as a lifesaving procedure, and 4 patients needed spinal surgery for stabilization with or without decompression, attributable to major spinal trauma. Patients were admitted for an average of 2.3 days in the ICU and an 8.5-days stay in the hospital, which has an inevitable burden on the healthcare system, as well as a significant treatment cost.

When we tried to determine the difference between operated and non-operated patients, the most prominent factor was GCS at admission, which was significantly lower in the operated group. None of the pedestrians needed surgery, and yet the fact that pedestrians were severely hit and needed hospitalization in the NICU or ER is a cause of distress and needs to be emphasized.

One more interesting fact was that most spinal injuries required surgery. It was significantly prominent compared to other mechanisms of injury.

Overall, operated patients spent significantly more time in the ICU and hospital, had significantly lower GOS-E at discharge and follow-up, and required significantly longer follow-up. It is important to underline that those patients were the more significantly injured; hence, the low GCS and longer stay was most likely not an effect of the surgery, but a consequence of a critical clinical status in the first place.

Generally, the outcome of e-bike related neurosurgical injuries was good. Most of the patients had a good recovery or were intact already at discharge. Those patients were only lightly injured, and yet we cannot ignore the fact that 3 patients died and 1 patient remained in a vegetative state. Also, at discharge, 6 patients were moderately to severely disabled, which has a significant impact on the patients and their family lives.

To stress the importance of helmets, patients who wore helmets had significantly higher GCS at admission; none of the patients presented with SAH or ICH, and, more important, none of them required for cranial surgery intervention. These patients spent less time in hospital and ICU

Given that the group of patients who wore helmets was extremely small, there was no difference in outcome between the groups.

We can freely assume that a patient who wore a helmet at the moment of trauma had much less neurosurgical trauma and therefore probably was not seen by our team and was not included in this research.

Patients with extremely severe injury and poorer outcome at discharge (severely to moderately disable) had a good chance for improvement by at least 1 point in the GOS-E at follow-up. Some patients who were discharged intact had worsened at the follow-up exam, mostly attributable to post-concussion syndrome issues or some post-spinal-injury disabilities that were not noted during the hospitalization. The most common symptoms patients complained about were headache, motor impairment, mood impairment, and also rehab status.

### Limitations

Our data are limited to patients admitted with cranial or cervical findings on imaging who were treated by our team. Certainly, some patients with minimal findings who were admitted to the ER and were treated only by ER medical staff were not included in this study. Other e-bike-related injuries as well as patients who were not transferred to medical care and those who were found dead at the scene were not included in this study. Because of the small number of patients who used a helmet, we could not assess the real impact that helmets have on injury patterns. Thus, our data are biased to patients who did not wear a helmet. However, a recent publication from our center, including all pediatric and adult ER admissions of e-bike injuries, stated that only around one fifth of all e-bike-related traumas used a helmet; thus, it is logical to deduce its value in e-bike use.

The second part of our data collection for this study was during the COVID-19 pandemic, including two lockdowns, which led to a significantly reduced number of active e-bikers and related trauma at this time; therefore, our data are underestimating the real numbers of e-bike-related injuries. Moreover, currently we witness a dramatical increase in e-bike-related neurosurgical trauma.

## Conclusion

This article describes our experience treating e-bike-related neurosurgical injuries in the adult population.

Although we could not estimate the total number of e-bike users, it occupies a huge volume of the total transportation. The unacceptable reality of the careless use and lack of education regarding this form of transportation mean leads to severe neurosurgical injuries, comorbidities, and even mortality. Our results reflect the risks of e-bikes in the adult population. Most of our patients were in the mid-age group, and almost none had used a helmet at the time of the accident. The results of this study highlight the potential need for surgical treatment, as well as the required admission duration in the ICU and regular departments, and the need for long-term rehabilitation and follow-up, reflecting the emotional and financial toll of these injuries.

This study emphasized both the need for proper regulation as well as an enforcement to reduce the number of injuries related to e-bikes. Once again, this study showed that helmets save lives and emphasized the importance of protecting our heads.

## Abbreviations Used

ASDH: acute subdural hematoma
DOS: duration of stay
EDH: epidural hematoma
ER: emergency room
GCS: Glasgow Coma Scale
GOS-E: Glasgow Outcome Scale-Extended
ICH: intracerebral hemorrhage
ICU: intensive care unit
IRB: institutional review board
NICU: neurosurgical ICU
RR: relative risk
SAH: subarachnoid hemorrhage
TBI: traumatic brain injury

## References

[1] Zhou SA, Ho AF, Ong ME, et al. Electric bicycle-related injuries presenting to a provincial hospital in China: a retrospective study. Medicine (Baltimore) 2017;96(26):e7395; doi: 10.1097/MD.0000000000007395 28658174 PMC5500096

[2] Karepov Y, Kozyrev DA, Benifla M, et al. E-bike-related cranial injuries in pediatric population. Childs Nerv Syst 2019;35(8):1393–1396; doi: 10.1007/s00381-019-04146-8 30989331

[3] Hermon K, Capua T, Glatstein M, et al. Pediatric electric bicycle injuries: the experience of a large urban tertiary care pediatric hospital. Pediatr Emerg Care 2020;36(6):e343–e345; doi: 10.1097/PEC.0000000000001395 29324633

[4] Papoutsi S, Martinolli L, Braun CT, et al. E-bike injuries: experience from an urban emergency department-a retrospective study from Switzerland. Emerg Med Int 2014;2014:850236; doi: 10.1155/2014/850236 24778880 PMC3979066

[5] Du W, Yang J, Powis B, et al. Epidemiological profile of hospitalised injuries among electric bicycle riders admitted to a rural hospital in Suzhou: a cross-sectional study. Inj Prev 2014;20(2):128–133; doi: 10.1136/injuryprev-2012-040618 23728530

[6] Gross I, Weiss DJ, Eliasi E, et al. E-bike-related trauma in children and adults. J Emerg Med 2018;54(6):793–798; doi: 10.1016/j.jemermed.2017.12.012 29352678

[7] Tenenbaum S, Weltsch D, Bariteau JT, et al.; Israeli Trauma Group. Orthopaedic injuries among electric bicycle users. Injury 2017;48(10):2140–2144; doi: 10.1016/j.injury.2017.08.020 28826652

[8] Weber T, Scaramuzza G, Schmitt KU. Evaluation of e-bike accidents in Switzerland. Accid Anal Prev 2014;73:47–52; doi: 10.1016/j.aap.2014.07.020 25173724

[9] Hu F, Lv D, Zhu J, et al. Related risk factors for injury severity of E-bike and bicycle crashes in Hefei. Traffic Inj Prev 2014;15(3):319–323; doi: 10.1080/15389588.2013.817669 24372505

[10] Baschera D, Jäger D, Preda R, et al. Comparison of the incidence and severity of traumatic brain injury caused by electrical bicycle and bicycle accidents—a retrospective cohort study from a Swiss Level I Trauma Center. World Neurosurg 2019;126:e1023–e1034; doi: 10.1016/j.wneu.2019.03.032 30857998

[11] Thompson DC, Rivara FP, Thompson R. Helmets for preventing head and facial injuries in bicyclists. Cochrane database Syst Rev 2000;1999(2):CD001855; doi: 10.1002/14651858.CD001855 10796827 PMC7025438

[12] Lee LK, Flaherty MR, Blanchard AM, et al.; Council on Injury, Violence, and Poison Prevention. Helmet use in preventing head injuries in bicycling, snow sports, and other recreational activities and sports. Pediatrics 2022;150(3):e2022058878; doi: 10.1542/peds.2022-058878 35965276

